# Common Single Nucleotide Polymorphisms in Genes Related to Immune Function and Risk of Papillary Thyroid Cancer

**DOI:** 10.1371/journal.pone.0057243

**Published:** 2013-03-08

**Authors:** Alina V. Brenner, Gila Neta, Erich M. Sturgis, Ruth M. Pfeiffer, Amy Hutchinson, Meredith Yeager, Li Xu, Cindy Zhou, William Wheeler, Margaret A. Tucker, Stephen J. Chanock, Alice J. Sigurdson

**Affiliations:** 1 Radiation Epidemiology Branch, Division of Cancer Epidemiology and Genetics, National Cancer Institute, National Institutes of Health, Department of Health and Human Services, Rockville, Maryland, United States of America; 2 Department of Head and Neck Surgery, The University of Texas M. D. Anderson Cancer Center, Houston, Texas, United States of America; 3 Biostatistics Branch, Division of Cancer Epidemiology and Genetics, National Cancer Institute, National Institutes of Health, Department of Health and Human Services, Rockville, Maryland, United States of America; 4 Core Genotyping Facility, SAIC-Frederick Inc., NCI-Frederick, Frederick, Maryland, United States of America; 5 Department of Epidemiology and Biostatistics, George Washington University, Washington D.C., United States of America; 6 Information Management Systems (IMS), Silver Spring, Maryland, United States of America; 7 Genetic Epidemiology Branch, Division of Cancer Epidemiology and Genetics, National Cancer Institute, National Institutes of Health, Department of Health and Human Services, Rockville, Maryland, United States of America; 8 Laboratory of Translational Genomics, Division of Cancer Epidemiology and Genetics, National Cancer Institute, National Institutes of Health, Department of Health and Human Services, Gaithersburg, Maryland, United States of America; Huazhong University of Science and Technology, China

## Abstract

Accumulating evidence suggests that alterations in immune function may be important in the etiology of papillary thyroid cancer (PTC). To identify genetic markers in immune-related pathways, we evaluated 3,985 tag single nucleotide polymorphisms (SNPs) in 230 candidate gene regions (adhesion-extravasation-migration, arachidonic acid metabolism/eicosanoid signaling, complement and coagulation cascade, cytokine signaling, innate pathogen detection and antimicrobials, leukocyte signaling, TNF/NF-kB pathway or other) in a case-control study of 344 PTC cases and 452 controls. We used logistic regression models to estimate odds ratios (OR) and calculate one degree of freedom *P* values of linear trend (*P_SNP-trend_*) for the association between genotype (common homozygous, heterozygous, variant homozygous) and risk of PTC. To correct for multiple comparisons, we applied the false discovery rate method (FDR). Gene region- and pathway-level associations (*P_Region_* and *P_Pathway_*) were assessed by combining individual *P_SNP-trend_* values using the adaptive rank truncated product method. Two SNPs (rs6115, rs6112) in the *SERPINA5* gene were significantly associated with risk of PTC (*P_SNP-FDR_/P_SNP-trend_ = *0.02/6×10^−6^ and *P_SNP-FDR_/P_SNP-trend_ = *0.04/2×10^−5^, respectively). These associations were independent of a history of autoimmune thyroiditis (OR = 6.4; 95% confidence interval: 3.0–13.4). At the gene region level, *SERPINA5* was suggestively associated with risk of PTC (*P_Region-FDR_/P_Region = _*0.07/0.0003). Overall, the complement and coagulation cascade pathway was the most significant pathway (*P_Pathway_* = 0.02) associated with PTC risk largely due to the strong effect of *SERPINA5*. Our results require replication but suggest that the *SERPINA5* gene, which codes for the protein C inhibitor involved in many biological processes including inflammation, may be a new susceptibility locus for PTC.

## Introduction

Thyroid carcinoma is the most common endocrine malignancy and its incidence has increased worldwide during the last 40 years [1]–[3]. Although improved diagnosis and reporting have likely contributed to this trend, additional reasons remain unclear [3]. Thyroid cancer is consistently more common in females, with a female-to-male ratio in incidence rates of about 3 to 1 [4]. One of the best established etiologic factors for thyroid carcinoma and its most common histological type, papillary thyroid cancer (PTC), is exposure to ionizing radiation during childhood [5], [6]. Family history studies suggest that thyroid cancer may have a greater familial component than other cancers with relative risk (RR) estimates of 3–4 or higher for a family history in first-degree relatives [7], [8]. Recent genome-wide association studies (GWAS) [9], [10] and a candidate gene study [11] have clearly implicated the gene *FOXE1*, formerly known as *TTF2* (thyroid transcription factor 2), as a susceptibility locus for PTC both in persons exposed [11] and unexposed to ionizing radiation [9], [11]; however, most genetic determinants of risk remain to be discovered.

Apart from childhood exposure to radiation and genetic predisposition, accumulating epidemiological, histological, and molecular evidence suggests that alterations in immune function might be related to risk of PTC [12], [13]. Chronic inflammation increasingly has been associated with many cancer types including PTC [12]–[14]. While the exact mechanism for these associations is unclear, it has been hypothesized that persistent inflammation leads to increased cellular turnover and provides selection pressure resulting in the emergence of cells that are at high risk for malignant transformation [14]. A history of autoimmune disease, in which inappropriate immune activation results in tissue destruction and inflammation, has also been associated with increased risk of PTC [15]. The most consistent associations for PTC are reported with a history of chronic autoimmune thyroiditis or Hashimoto thyroiditis (HT) [16], [17], both more common in females [4], [18]. At a histological level, HT and PTC share lymphoid infiltration, expression of galectin 3, CITED1, cytokeratin 19, and fibronectin 1 through which the association might be mediated [12], [16]. At the molecular level, activation of proinflammatory MAPK-signaling pathway through *RET/PTC* rearrangement might contribute to development of HT-associated PTC [12], [17]. Recently common germline polymorphisms in immune-related genes have been associated with risk of many autoimmune diseases, including HT [19], [20], but such data in relation to PTC are sparse.

Given emerging relationships between immune dysfunction and PTC, we sought to identify genetic susceptibility markers for PTC in a case-control study of 344 PTC cases and 452 controls by evaluating 3,985 tag single nucleotide polymorphisms (SNPs) in 230 candidate gene regions involved in various immune pathways (adhesion-extravasation-migration, arachidonic acid metabolism/eicosanoid signaling, complement and coagulation cascade, cytokine signaling, innate pathogen detection and antimicrobials, leukocyte signaling, TNF/NF-kB signaling and other). This effort extends previous candidate gene based studies of PTC by increasing the number and coverage of promising genes implicated in the development of inflammation, autoimmunity, and carcinogenesis.

## Materials and Methods

### Study Population

Cases included thyroid cancers (n = 202) diagnosed within the U.S. Radiologic Technologists (USRT) cohort as described previously [21] and cancers (n = 142) diagnosed and treated at the University of Texas M. D. Anderson Cancer Center (UTMDACC) [22]. All cases were histologically confirmed. Controls (n = 452) were selected from the USRT study [23] and frequency-matched to the USRT cases by sex and year of birth (+/−2 years). These controls were also used for the combined case series (USRT and UTMDACC). Both studies were reviewed and approved by the Special Studies Institutional Review Board of the National Cancer Institute and Institution Review Board of the University of Texas M. D. Anderson Cancer Center, respectively. All subjects provided written informed consent.

To minimize the potential for population stratification and phenotypic heterogeneity of thyroid cancer cases, we excluded individuals with non-European ancestry (n = 97) and cases with follicular thyroid cancer (n = 17) leaving 344 papillary thyroid cancer cases (n = 202 USRT and n = 142 UTMDACC) and 452 controls of European ancestry with validated genotyping results. Allele frequencies for papillary thyroid cancer cases of European ancestry were largely similar between the USRT and the UTMDACC study sites and between males and females, so these groups were combined for genetic analyses.

### Variable Collection and Data Harmonization

Data concerning demographics, health history, family history of cancer, and other risk factors were collected by self-administered mailed questionnaires or telephone interview in the USRT study or a self-administered questionnaire at the time of blood collection in the UTMDACC study. Race, histological type of thyroid cancer, and history of autoimmune thyroiditis were defined similarly in both studies. Other variables were harmonized across the two studies (cigarette smoking, alcohol consumption, prior exposure to therapeutic radiation, and family history of any cancer or specifically of thyroid cancer). Because controls were matched to cases on year of birth (+/−2 years), and to enable time-appropriate partitioning of exposures (such as cigarette smoking), we assigned a referent age to controls to correspond to the time of a case’s diagnosis. Specifically, a control for a particular case was randomly selected from case-defined strata of sex and year of birth. If no control could be found then matching on sex and year of birth was relaxed (in that order). The algorithm was repeated until referent age was assigned to every control. Time dependent exposures in controls were accounted for up to the referent age and ignored thereafter.

### Laboratory Methods

#### DNA extraction

In the USRT study, venipuncture whole blood samples were shipped with a temperature stabilizing pack overnight to the processing laboratory in Frederick, MD. At UTMDACC, venipuncture whole blood samples were collected and processed in the clinic. Both studies extracted DNA from peripheral blood leucocytes using Qiagen Mini Kits (Qiagen Inc., Valencia, CA) according to the manufacturer’s instructions.

#### Genotyping

Genotyping of 27,904 SNPs, including tag SNPs in 1,316 candidate genes and their surrounding regions (within 20 kb 5′ of the start of transcription at the first exon and 10 kb 3′ of the last exon) and intergenic SNPs included on the platform because of associations with several cancers, was performed at the NCI Core Genotyping Facility (Advanced Technology Center, Gaithersburg, MD; http://cgf.nci.nih.gov/) using the custom-designed iSelect Infinium assay (Illumina, www.illumna.com). Tag SNPs were selected for target gene regions from the common SNPs (minor allele frequency, MAF >5%) genotyped by the HapMap Project (Data Release 20/Phase II, NCBI Build 36.1, assembly dbSNPb126) in the Caucasian population (CEU) using TagZilla software (http://tagzilla.nci.nih.gov/) based on the pairwise binning method of Carlson et al. [24] with a binning threshold of r^2^>0.8.

#### Quality control

Out of 27,904 SNPs included in the genotyping platform, 722 failed genotyping (no amplification or clustering) and 208 had monoallelic calls and were excluded from the analysis. SNPs with <95% concordance (n = 656) or <90% completion (n = 740) among randomly inserted quality control replicates, and 740 SNPs that failed the Hardy-Weinberg Equilibrium test in controls (*P* value <0.00001) were also excluded. Of the 947 study participants with DNA specimens (n = 232 USRT cases, n = 223 UTMDACC cases, n = 492 USRT controls), we excluded subjects if their samples failed genotyping (n = 18), were not assayed (n = 4), or had less than 90% completion rate (n = 15).

### Final Set of Tag SNPs, Sub-pathways, and Analytic Population

Following quality control related exclusions, we also excluded in the analysis 1,607 intergenic SNPs that had been previously implicated in etiology of non-thyroid cancers, and 5,706 tag SNPs with the MAF <10% or the lowest achievable significance level computed from the marginal totals >1^−30^ [25]. After all exclusions, there were 17,525 tag SNPs in 1,129 gene regions available for analysis. Of these, 3,985 SNPs were in 230 gene regions involved in immune-related pathways that are the subject of the current analysis. The candidate gene regions were further subdivided into eight *a priori* defined pathways (adhesion-extravasation-migration, arachidonic acid metabolism/eicosanoid signaling, complement and coagulation cascade, cytokine signaling, innate pathogen detection and antimicrobials, leukocyte signaling, TNF/NF-kB signaling, and other) largely based on classifications from Loza et. [26]. When assigning gene regions with a broad range of known functions, we allowed for allocation to multiple pathways. A complete list of the 230 gene regions and their allocation to the respective pathways is available in Table S1.

### Statistical Analysis

We organized the analytic approach by first examining the relationship of individual SNPs with PTC risk, followed by examining relationships at the gene region level, pathway level, and overall to also detect small SNP effects that could only be detected in the aggregate.

#### SNP-based associations

Logistic regression models were used to estimate odds ratios (ORs) and to calculate 95% confidence intervals (CIs) of the association of PTC risk with each SNP genotype, coded as 0, 1, 2, with 0 through 2 denoting the number of minor alleles. We calculated the linear *P_SNP-trend_* for SNP genotype in crude models and models adjusted for sex, attained age in four categories (<35, 35–44, 45–54, 55+ years), and year of birth (<1940, 1940–1949, 1950+) as an ordinal variable. The ORs from models with and without adjustments were very similar, so we present the results from the adjusted models. For the top SNPs (*P_SNP-trend_* <0.001), we repeated analyses of main effects while also (1) adjusting for history of autoimmune thyroiditis, (2) limiting to individuals without autoimmune thyroiditis, and (3) limiting to females. For the top SNPs we also conducted interaction analyses with attained age and history of autoimmune thyroiditis. These analyses were based on one degree of freedom likelihood ratio tests comparing the fit of two models: (1) including main effect of genotype (0, 1, 2) and continuous age or history of autoimmune thyroiditis (0, 1), and (2) including also the multiplicative interaction term between the genotype and age or autoimmune thyroiditis. To correct for multiple comparisons, we used the false discovery rate (FDR) control method [27].

#### Gene region- and pathway-based analyses

We combined SNP specific *P* values of linear trend within the same gene region (*P_Region_*) using an adaptive rank truncated product (ARTP) method [28]. This method accounts for the linkage disequilibrium (LD) structure within the gene region and accounts for the number of SNPs included in the *P* value calculation. For the subset of genes with *P_Region_* <0.01, we evaluated pairwise indices of LD (D′ and r^2^) in controls using the Haploview package [29]. Gene region-level *P* values were further combined into the *P* values for pathways (*P_Pathway_*) and an overall P value for the entire group of immune genes (*P_Overall_*) using the ARTP method. Pathway-based analyses were repeated including and excluding gene regions with multiple allocations.

Statistical analyses were conducted in SAS version 9.1 (SAS Institute, Cary, NC) and in *R*, unless otherwise noted.

## Results

The characteristics of 344 PTC cases and 452 controls are summarized in Table 1. There was a lower proportion of females among cases compared to controls (79.7% vs. 93.6%) owing to the fact that study controls were originally selected from the USRT cohort to match the USRT cases (90.6% female), but subsequently were used for the UTMDACC cases containing a lower proportion of females (64.1%). The distribution of referent age in cases and controls was comparable. After controlling for sex and age, cases were less likely to smoke (*P*<0.001) or to have several family members with a history of cancer (*P = *0.009). Cases were also less likely than controls to drink alcohol, although this difference was not statistically significant (*P* = 0.073), but more likely to have at least one family member with a history of thyroid cancer (*P = *0.006), a higher body mass index (*P*<0.001), and a personal history of autoimmune thyroiditis (*P*<0.001). The adjusted OR associated with a history of autoimmune thyroiditis was 6.4 (95% CI: 3.0–13.4).

**Table 1:** Characteristics of the study population by case and control status, U. S. Radiologic Technologists Study and University of Texas M. D. Anderson Cancer Center Study.

	Cases (N = 344)	Controls (N = 452)	
Characteristic	n (%)	n (%)	*P* a
**Study**			
Radiologic Technologists	202 (58.7)	452 (100.0)	
M. D. Anderson Cancer Center	142 (41.3)	0	
**Gender**			<0.001
Female	274 (79.7)	423 (93.6)	
Male	70 (20.3)	29 (6.4)	
**Attained/referent age, year**			0.360
19–25	26 (7.6)	30 (6.6)	
26–35	73 (21.2)	103 (22.8)	
36–45	113 (32.8)	159 (35.2)	
46–55	77 (22.4)	105 (23.2)	
56–65	43 (12.5)	49 (10.8)	
66–79	12 (3.5)	6 (1.3)	
**Smoking status** b			<0.001^c^
Never	208 (60.5)	234 (51.8)	
Former	87 (25.3)	101 (22.4)	
Current	48 (14.0)	113 (25.0)	
Unknown	1 (0.3)	4 (0.9)	
**Alcohol consumption** b			0.073^c^
Never	179 (52.0)	213 (47.1)	
Ever	158 (45.9)	221 (48.9)	
Unknown	7 (2.0)	18 (4.0)	
**Number of relatives with cancer** b			0.009^c^
None	170 (49.4)	171 (37.8)	
One	107 (31.1)	180 (39.8)	
Two	39 (11.3)	64 (14.2)	
Three or more	18 (5.2)	33 (7.3)	
Adopted	0	4 (0.9)	
Unknown	10 (2.9)	0	
**Number of relatives with thyroid cancer** b			0.006^c^
None	319 (92.7)	442 (97.8)	
At least one	15 (4.4)	6 (1.3)	
Adopted	0	4 (0.9)	
Unknown	10 (2.9)	0	
	**Mean (sd)**	**Mean (sd)**	
**BMI, kg/m^2b^**	26.1 (6.0)	24.4 (4.7)	<0.001^c^
**History of autoimmune thyroiditis** b			
No	277 (80.5)	432 (95.6)	<0.001^c^
Yes	48 (14.0)	9 (2.0)	
Unknown	19 (5.5)	11 (2.4)	

a Test of independence.

b As of referent age. See text for description of referent age assignment.

c Adjusted for sex and attained age.

### SNP-based Associations

**Figure 1:**
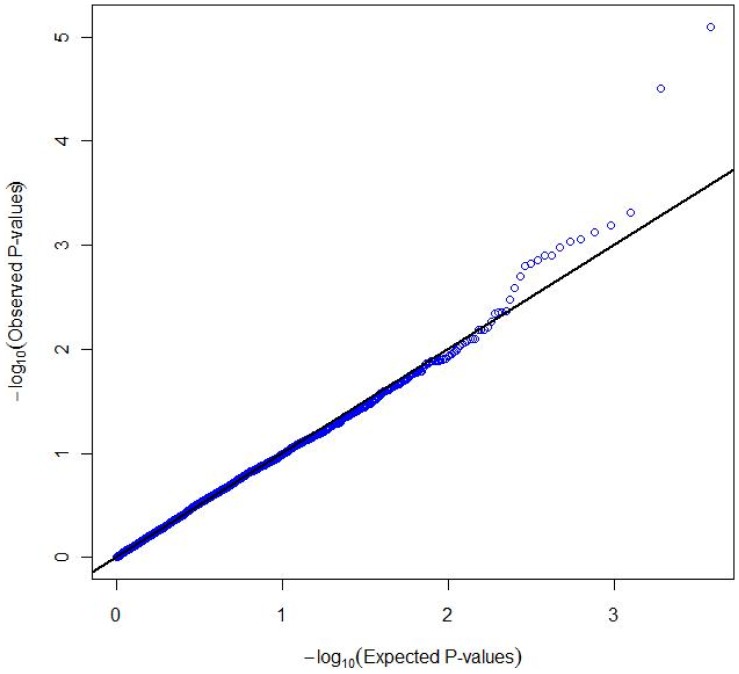
Observed and expected distributions for the P values of linear trend for 3,985 tag-SNPs in 230 gene regions related to immune function in a case-control study of PTC risk.

**Figure 2:**
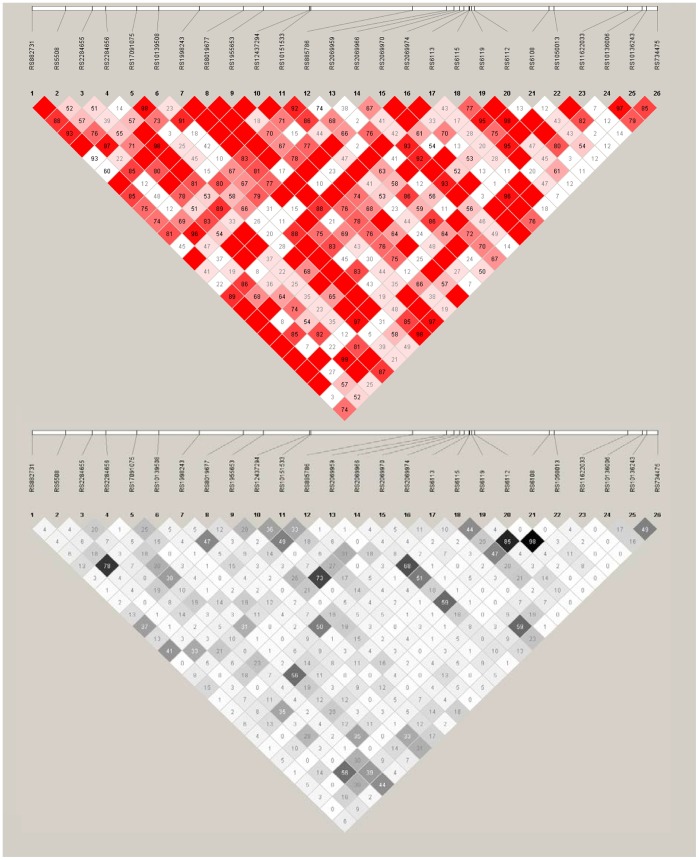
Linkage disequlibrium (LD) representation of the *SERPINA5* gene. The figure represents LD based on the genotyping data from the study controls with European ancestry. LD is measured by D′ parameters (top panel) and the r^2^ correlation coefficient (bottom panel). D′ and r^2^ values of one are interpreted as complete LD.

While the observed distribution of *P* values of linear trend for all 3,985 SNPs was not statistically different from the expected uniform (null) distribution, there was some suggestion of departure from the null in the area of lowest *P* values (Figure 1). Seven SNPs in four gene regions were associated with risk of PTC at *P_SNP-trend_* <0.001 (Table 2; a complete list of all *P_SNP-_*_trend_ values is available in Table S2). Of these, four SNPs (rs6115, rs6112, rs6108, rs10139508) were in the *SERPINA5* gene and the remaining SNPs were in the *MASP1* (rs850316), *HEMGN* (rs10984462), and *TICAM1* (rs2292151) genes, respectively. Two SNPs (rs6115, rs6112) remained significant after FDR correction (*P_SNP-FDR = _*0.02 and *P_SNP-FDR = _*0.04, respectively); both were in *SERPINA5*. The LD structure for the *SERPINA5* gene is presented in Figure 2. There is evidence that the two most significant SNPs (rs6115 and rs6112) are in LD with one another (D′ = 0.95 and r^2^ = 0.47). The associations with the top SNPs were not meaningfully changed when analyses were adjusted for history of autoimmune thyroiditis or restricted to individuals without history of autoimmune thyroiditis or conducted only among females (data not shown). Also, there was no evidence that these associations significantly varied by attained age or history of autoimmune thyroiditis (data not shown).

**Table 2:** Odds ratio and 95% confidence interval (CI), adjusted for sex, attained age, and year of birth, for the tag SNPs associated with papillary thyroid cancer risk at *P_SNP-trend_* <0.001.

			Cases	Controls			
Gene Region	SNP	Genotype	n (%)	n (%)	Odds Ratio	95% CI	*P_SNP-trend_* a/*P_SNP-FDR_* b
*SERPINA5*	rs6115	AA	119 (35.2)	228 (51.5)	1.00	Referent	
		AG	148 (43.8)	161 (36.3)	1.68	1.20–2.34	
		GG	71 (21.0)	54 (12.2)	2.62	1.68–4.08	6.08*10^−6^/0.02
*SERPINA5*	rs6112	CC	120 (34.9)	227 (50.2)	1.00	Referent	
		CT	182 (52.9)	196 (43.4)	1.72	1.25–2.36	
		TT	42 (12.2)	29 (6.4)	2.72	1.56–4.75	2.23*10^−5^/0.04
*SERPINA5*	rs6108	TT	107 (31.3)	187 (41.6)	1.00	Referent	
		AT	173 (50.6)	218 (48.4)	1.34	0.96–1.86	
		AA	62 (18.1)	45 (10.0)	2.54	1.56–4.12	0.00028/0.37
*SERPINA5*	rs10139508	AA	118 (34.4)	208 (46.0)	1.00	Referent	
		AG	183 (53.4)	203 (44.9)	1.74	1.26–2.39	
		GG	42 (12.2)	41 (9.1)	1.90	1.12–3.20	0.00075/0.50
*MASP1*	rs850316	GG	79 (23.0)	157 (34.7)	1.00	Referent	
		AG	180 (52.3)	215 (47.6)	1.67	1.17–2.38	
		AA	85 (24.7)	80 (17.7)	2.04	1.32–3.15	0.00082/0.50
*HEMGN*	rs10984462	TT	216 (63.0)	246 (54.4)	1.00	Referent	
		GT	114 (33.2)	166 (36.7)	0.72	0.52–0.99	
		GG	13 (3.8)	40 (8.8)	0.35	0.18–0.68	0.00087/0.50
*TICAM1*	rs2292151	GG	182 (52.9)	283 (63.0)	1.00	Referent	
		AG	133 (38.7)	145 (32.3)	1.50	1.09–2.07	
		AA	29 (8.4)	21 (4.7)	2.34	1.25–4.38	0.00088/0.50

a SNP-based linear *P_SNP-trend_* is calculated based on the three-level genotype (0, 1, and 2) in logistic regression models adjusted for sex, attained age, and year of birth.

b False discovery rate (FDR) corrected linear *P_SNP-trend_*.

### Gene Region-based Associations

Largely consistent with individual SNP analyses, four gene regions (*SERPINA5*, *HEMGN, TICAM1,* and *FCGR2A*) were associated with PTC risk at *P_Region_* <0.01 (Table 3). After multiple comparisons adjustment, only *SERPINA5* remained suggestively associated with PTC risk at *P_Region-FDR = _*0.069. All gene region-based results are available in Table S3.

**Table 3:** Significance levels (*P* values) for an association with papillary thyroid cancer for the selected gene regions with uncorrected *P_Region_* <0.01.

GeneRegion	ChromosomeBand	Number ofSNPs	*P_Region_* a */P_Region-FDR_* b
*SERPINA5*	14q32.1	26	0.0003/0.069
*HEMGN*	9q22.33	5	0.0016/0.183
*TICAM1*	19p13.3	15	0.0066/0.426
*FCGR2A*	1q23	8	0.0074/0.426

a Gene region-based *P* values (*P_Region_*) calculated using the adaptive rank truncated product method.

b False discovery rate (FDR) corrected *P_Region_*.

### Pathway-based Associations

Two immunity pathways, complement and coagulation cascade including the *SERPINA5* gene and TNF/NF-kB signaling pathway including the *HEMGN* gene, were significantly associated with risk of PTC (*P_Pathway_ = *0.02 including and *P_Pathway_ = *0.02 excluding overlap; and *P_Pathway_ = *0.05 including and *P_Pathway_ = *0.04 excluding overlap, respectively) (Table 4). The immunity pathway as a whole was not statistically significantly associated with PTC risk.

**Table 4:** Pathway-based significance levels (*P* values) for each immunity pathway analyzed, with and without accounting for overlapping genes between pathways.

	With	Overlap	Without	Overlap
Pathway	*P_Pathway_* b	Number of Genes	*P_Pathway_* b	Number of Genes
Adhesion-extravasation-migration	0.82	33	0.80	30
Arachidonic acid metabolism/eicosanoid signaling	0.56	20	0.56	20
Complement and coagulation cascade	0.02	25	0.02	25
Cytokine signaling	0.90	75	0.85	63
Innate pathogen detection and antimicrobials	0.15	46	0.16	41
Leukocyte signaling	0.23	20	0.20	14
TNF/NF-kB signaling	0.05	29	0.04	13
Other	0.07	7	0.08	7
Overall	0.22	235	0.18	212

a Some gene regions were allocated to more than one pathway, creating overlap. To calculate *P* values without overlap in multiple pathway allocations, these specific gene regions were dropped from their respective pathways.

b Pathway-based *P* values (*P_Pathway_*) were calculated using the adaptive rank truncated product method.

## Discussion

We evaluated the associations of 3,985 tag SNPs in 230 candidate genes from several immune pathways in relation to risk of PTC. The gene collection overall was not significantly associated with risk of PTC. In pathway-based analyses, the complement and coagulation cascade and TNF/NF-kB signaling pathway were significantly associated with risk of PTC. These findings were largely attributed to the effects of *SERPINA5* and *HEMGN* genes, respectively. At the SNP level, the strongest associations were found for two SNPs in the *SERPINA5* gene (rs6115 and rs6112), both of which persisted after FDR correction and were independent of history of autoimmune thyroiditis. While our findings require replication, they strongly suggest that the *SERPINA5* gene region might be a novel susceptibility locus for PTC.

The main findings in our study are internally consistent in that at the SNP, gene region, and pathway level they are attributed to the *SERPINA5* gene. This gene is located on human chromosome 14 at position 14q32.1 and codes the protein C inhibitor (PCI), a member of the plasma serine protease inhibitor family [30]. While originally it was discovered as an inhibitor of activated protein C, today PCI is known to play a role in many biological processes beyond hemostasis and thrombosis including inflammation, innate immunity, carcinogenesis, and fertilization [30]–[33]. Recent proteomic analysis of human multiple sclerosis lesions by sensitive mass spectrometry implicated PCI in the development of chronic active plaques and suggested an unexpected intersection between coagulation and inflammation [34]. The expression of *SERPINA5* was found to be significantly reduced in advanced-stage serous ovarian borderline tumors and serous carcinomas when compared with the early-stage counterparts, and such a reduction was linked to more aggressive features of the ovarian borderline tumors [35]. We were unable to find any reports of changes in *SERPINA5* expression in thyroid cancer, although several studies reported over-expression in *SERPINA1* (belonging to the same gene cluster on human chromosome 14q32.1) in sporadic and radiation-related thyroid cancers [36], [37]. The relationship of over-expression in *SERPINA1* to *SERPINA5* SNPs is unclear and needs to be interpreted cautiously. To our knowledge, there have been no reports of germline polymorphisms in *SERPINA5* in relation to risk of thyroid cancer or any other cancer, but there have been several reports of associations with the rate of fertilization failure [38] or risk of Wegener’s granulomatosis [39], an autoimmune vasculitis syndrome. It is intriguing that one of the two linked SNPs in *SERPINA5* gene (rs6115), which withstood correction for multiple comparisons, results in a serine to asparagine amino acid change at position 64. However, whether the observed positive association between rs6115 polymorphism and risk of PTC is due to changes in PCI properties related to amino acid substitution or to potential LD with another unidentified variant remains unknown and requires further evaluation.

The second strongest association found in our study concerned SNPs in the *HEMGN* gene, although these did not withstand correction for multiple comparisons at the individual SNP and gene level. Interestingly, the *HEMGN* gene is located on chromosome 9q22.33 within a LD region including the *FOXE1* gene necessary for maintenance of the differentiated state of the thyroid [40]. Several recent GWAS and a parallel candidate gene study reported consistent associations between variants in the *FOXE1* region (rs965513, rs1867277, rs7850258) and risk of papillary thyroid cancer and/or primary hypothyroidism [9]–[11], [41], [42]. Unfortunately, the *FOXE1* gene was not included in our genotyping platform, but due to extensive linkage in the area, signal associated with *HEMGN* is unlikely to be independent and is in accord with previously reported associations for variants in *FOXE1*.

In interpreting the results of our study, several strengths and limitations need to be considered. Because survival rates for PTC are exceptionally high, any associations that might be due to survival bias are unlikely. Our study had high participation rates, minimizing potential for selection bias. To minimize concerns about population stratification, all analyses were limited to individuals of European ancestry. Moreover, cases from the two studies were similar with regard to age at diagnosis, smoking status, tumor size, and frequency of many alleles. Other strengths of our study include a thorough selection of genes related to a variety of immunity pathways. The proportion of females in the UTMDACC cases was lower than in the USRT cases and respective controls; to assure that the observed associations are not confounded, analyses were adjusted for sex. Results based on adjusted analysis as well as on analysis restricted to females were not meaningfully different. Similarly, the observed associations appeared to be independent of history of autoimmune thyroiditis. Relative to GWAS, the coverage of selected gene regions was higher, although we could have missed important associations with SNPs in gene regions not included within the genotyping platform such as in *FOXE1*. While among the larger studies in the field with respect to the number of thyroid cancer cases and controls, our study had limited power (<80%) to detect modest-to-weak associations (OR <1.7) especially for less common genetic variants (MAF ≤10%). Another limitation is the use of tag SNPs that are themselves unlikely to be the disease-related SNPs, but are assumed to be in LD with the causal variant. To address these limitations, we excluded SNPs with MAF <10% and relied on robust gene/pathway ARTP methods combining SNP-specific *P* values of linear trend to confirm associations with risk of PTC. Although we targeted genes with strong prior evidence and adjusted for multiple comparisons using the FDR method, we cannot exclude the possibility of false positives.

In summary, we identified a promising susceptibility locus for PTC in the *SERPINA5* gene belonging to the complement and coagulation cascade pathway and confirmed previously reported associations with polymorphisms in the *FOXE1 - HEMGN* region. Subsequent replication and fine mapping studies with adequate power are essential to establish causal variants in the *SERPINA5* region.

## Supporting Information

Table S1 **Gene regions and their allocation to the respective pathways: U. S. Radiologic Technologists Study and University of Texas M. D. Anderson Cancer Center Study of papillary thyroid cancer.**
(XLS)

Table S2 ***P*** **values of linear trend for 3,985 SNPs in immune related genes: U. S. Radiologic Technologists Study and University of Texas M. D. Anderson Cancer Center Study of papillary thyroid cancer.**
(XLS)

Table S3 **Gene region-based** ***P*** **values for an association with thyroid cancer: U. S. Radiologic Technologists Study and University of Texas M. D. Anderson Cancer Center Study of papillary thyroid cancer.**
(XLS)
